# Examining communalism in the home math environment to understand its role in predicting children’s mathematics development

**DOI:** 10.3389/fpsyg.2025.1582091

**Published:** 2025-09-10

**Authors:** Tamika L. McElveen, Annahita Modirrousta, Carlie Fox, John Day, Sophie Salerno, Abby Murchland

**Affiliations:** Psychology, Miami University, Oxford, OH, United States

**Keywords:** home math environment, math skill development, communal beliefs, factor structure, mediation analysis

## Abstract

This study investigated the structure of the home math environment (HME) of preschoolers. Examinations include the relation between parental communal beliefs, home math engagement, children’s math skill development, and whether communal beliefs mediated these relations. Parents reported data (*N* = 652, 49% female, mean age = 4.25; parental demographics: 87.4% White, 9% Latine and Hispanic, 6% African American, 58% 4-year degree or higher, mean income = $70,000–$79,999). The HME structure diverged from prior work. Significant differences were found in home math engagement based on children’s age. Communal beliefs were significantly different based on parental ethnicity and education. While children’s HME and communal beliefs were significantly associated with their math skill development, communal beliefs were not a significant mediator. Overall, this study provided important progress toward understanding the role of these constructs in the development of children’s early math skills.

## Introduction

The home mathematics environment plays a crucial role in shaping preschoolers’ mathematics skill development, with various factors influencing children’s early numeracy abilities (Daucourt et al., 2021). In turn, the development of early numeracy skills has significant implications for children’s later mathematics achievement (Nguyen et al., 2016; Watts et al., 2014). The home mathematics environment consists of families engaging children in direct and indirect mathematical activities, such as counting, puzzles, and everyday problem-solving tasks (Hart et al., 2016). Race, ethnicity, gender, and socioeconomic status have been identified as important demographic and contextual factors in the home mathematics environment (Eason et al., 2022; Elliott and Bachman, 2018; Sonnenschein and Dowling, 2019). Sociocultural factors such as communal values can be important contributors as well. *Communal value* refers to a belief in social connectedness and social interdependence that leads to aims such as helping people and working collaboratively with others (Dasgupta et al., 2022; McElveen, 2024).

Communal value has been identified as positively contributing to mathematics learning and achievement in primary, secondary, and postsecondary contexts (Boykin and Bailey, 2000; Brown et al., 2015; Gray et al., 2020). Extending the examination of communal value in the home mathematics environment is a wider and more inclusive developmental lens to examine its association with the development of early mathematics skills. In theory, communal value is a cultural asset that guides the learning process as families use culturally relevant strategies such as shared and collaborative learning experiences to scaffold children’s math skill development (Coleman et al., 2023). Therefore, exploring the practical implications of communal value as a developmental asset for preschoolers and their families can inform the interventions that are communicated to families, particularly those from historically minoritized backgrounds.

This study examined the influence of the families’ engagement and sociocultural factors on children’s early mathematics (math) skill development. First, we examined the direct effect of the home learning environment and parents’ communal value on preschoolers’ math skill development. Next, we examined the mediating effect of communal value on the relation between the home learning environment and math skill development. Finally, we discuss the theoretical and practical implications of this approach.

### Early math skills development

Early math skill development has implications for children’s math achievement as they enter elementary school as well as their academic achievement in primary and secondary school (Duncan et al., 2007; Jordan et al., 2009; Rittle-Johnson et al., 2017; Watts et al., 2014). Parents, caregivers, and families play a critical role by fostering an environment that contributes to early math skills development through the (1) availability of math-related resources at home, including books, puzzles, and educational toys to enrich children’s exposure to math concepts and promote active exploration and discovery, (2) socialization of math-related values, beliefs, and expectations, (3) engagement with schools to support children’s learning, and (4) social interactions within the home that engage children in learning math concepts and skills (math home environment).

### Categorization and measurement of early math skills

Assessing math skills in preschool children involves identifying key factors in their home learning environment and understanding the diverse ways in which young learners engage with mathematical concepts. One of the more general ways to categorize the measures is by using the terms “formal home learning environment” and “informal learning environment” to describe early numeracy skill development. The formal home learning environment is characterized by direct learning activities that teach early numeracy skills, including counting or magnitude understanding, set comparison, number identification, adding and subtracting, and patterning. The informal learning environment offers indirect learning opportunities, such as playing games or other activities that require the processing of numbers and the development of numerical strategy (Gashaj et al., 2023). Activities within the formal (e.g., operational activities involving the manipulation of numbers or quantities) and informal (e.g., shared number game play) learning environment have predicted children’s growth in numeracy outcomes and applied problem-solving skills (Susperreguy et al., 2020).

Measurements of the home math environment seek to capture the developmental trajectory of children’s early math skills. Whereas verbal rote counting may be practiced as early as 2 years old through the introduction of math-related songs, cardinality (i.e., the recognition that the last number counted represents the total number of a set) may be developed between after the age of three (Mutaf-Yıldız et al., 2020). By assessing the frequency of formal and informal learning experiences, scholars gain insight into the variations in engagement across home math activities (Ehrman et al., 2023; Hart et al., 2016). Frequency of activities that fostered mathematical skills has also predicted children’s math performance during their early school years (LeFevre et al., 2009). In addition, assessment of children’s difficulty when engaging in various formal and informal math activities provides further insight into parents’ perceptions of the difficulty of each home math activities. Findings related to the difficulty of specific home math activities have identified activities that increase (e.g., measuring length and width), decrease (e.g., counting objects), or remain consistent (e.g., playing with blocks) over time (Ehrman et al., 2023).

### Beyond numeracy

Over the past two decades, exploration of the home math environment’s measurement structure has revealed a conceptual expansion to include spatial activities (Dearing et al., 2012; Hart et al., 2016; Purpura et al., 2020) and patterning (Rittle-Johnson et al., 2019). While the literature surrounding the Home Mathematic Environment (HME) has been steadily increasing, there is a noticeable lack of research surrounding the role of non-numeracy skills, such as patterning and spatial skills, in mathematics development (Hornburg et al., 2021). There is evidence that numeracy and non-numeracy skills are related to mathematical and problem-solving skill development, with longitudinal and causal evidence supporting their role in math knowledge (Rittle-Johnson et al., 2019).

### Social and cultural factors influencing early math skills development

Among the social and cultural factors present in the home math environment that influence children’s early math skills development (i.e., socioeconomic status, gender, race, and ethnicity), socioeconomic status is heavily researched, with mixed results. Higher parental education and parental income have been associated with more indirect math activities and early math skill development (Elliott and Bachman, 2018; Susperreguy et al., 2020). Further, the connection between parental involvement, socioeconomic status, and elementary students’ mathematical achievement has shown a strong, positive correlation (Alghazo and Alghazo, 2015). On the other hand, studies have found that even after controlling socioeconomic background, parent–child performance was positively correlated with parental cumulative number talk and number skills (Elliott and Bachman, 2018; Missall et al., 2015).

Beyond socioeconomic status, variations in children’s gender, race, and ethnicity may also have implications in the home mathematics environment and for children’s math skills development. Though a few studies have found differences in math engagement based on gender, race, and ethnicity (Eason et al., 2022), these factors are not consistently and explicitly analyzed and discussed in the home mathematics literature. Utilizing prior research focused on adult and adolescent populations, social orientation (communal vs. individualism) plays a significant role in health- and academic-related outcomes beyond considerations of race, ethnicity, and socioeconomic status (Abdou et al., 2010; Hurley et al., 2023).

Social orientation refers to one’s approach to social interactions influenced by cultural values, social norms, heritage, and traditions cultivated within one’s home and community (Abdou et al., 2010; American Psychological Association, Task Force on Resilience and Strength in Black Children and Adolescents, 2008; Boykin, 1986). It is conceptually distinct due to its focus on individualistic or independent vs. communal or interdependent perspectives and behaviors. While self-direction and autonomy are endorsed in families with an individualistic orientation, communally oriented families emphasize relatedness and connection (Varnum et al., 2010). Recognizing that learning involves the interdependence of activities, concepts, and culture (Brown et al., 1989), understanding the role of a communal orientation could provide insights into the home mathematics environment. As an example, Coleman et al. (2017) measured elementary students’ perceptions of communal activities and attitudes in their home environments during their investigation of communal learning and math performance. They found a positive correlation between home communal orientation and children’s mathematics performance on fraction identification leading to an indication of the role of social orientation on the foundation of children’s conceptual knowledge.

The importance of communal learning has been substantiated in various contexts, including early literacy environments (Sénéchal and LeFevre, 2002; Sim et al., 2014) and early math environments, which predicted children’s growth on nonsymbolic comparison tasks, arithmetic fluency, and applied problem-solving (Susperreguy et al., 2020). Consistently, research in school contexts has found that when students were placed in individualistic vs. communal learning conditions, those who participated in communal learning outperformed on mathematics assessments (Boykin and Bailey, 2000; Coleman et al., 2017; Hurley et al., 2023).

### The current study

Ultimately, many unanswered questions still remain regarding the development of math skills in young children. The body of literature surrounding the Home Math Environment is still relatively small, but as this continues to grow, research highlights the need for the identification of factors that may influence HME, best methodological designs, and characterization of how primary caregivers incorporate mathematical activities into the home environment (Hornburg et al., 2021). The inclusion of other non-numerical domains in the HME may also be important in identifying the key areas of mathematical development during early childhood, as well as acknowledging the impact caregiver understanding and self-reporting can have on research results. A broader examination of math skills and experiences in the home context is also needed, specifically in parent–child exploration of math skills (Zippert et al., 2020). Social orientation as a sociocultural context is an avenue to explore in researching math skills development and the HME. Increasing our understanding of how the HME impacts mathematical skill development can be crucial for young children both academically and beyond, offering insights into how primary caregivers may enhance mathematical learning and how teachers and curriculum may reflect these results as they are incorporated into the classroom setting as well.

To address these unanswered questions, the current study examined the mediating role of parental communalism on the relation between Home Math Environment and Child Math Skills Development. We conducted several steps in the analysis prior to answering this research question. First, we examined the factor structure of the home math environment to conceptualize the practice of home math teaching and learning for this sample population. Next, we conducted preliminary analyses to understand each construct separately (i.e., communalism, home math activities, and math skills), as well as the relation between communalism and the home math environment with children’s math skills development. Finally, we examined the mediating role of communalism in the relation between the home math environment and children’s math skills development. Our hypothesis is a positive and significant relation between children’s home math environment and their math skills development. We also expect communalism to mediate this relation. Related to the home math environment, we examined the frequency and difficulty of home math activities as both have empirical support and importance (Ehrman et al., 2023; Mutaf-Yıldız et al., 2020).

## Materials and methods

### Participants

Data was collected from parents via a parent report through the online platform Prolific (Ellis et al., 2022). Participants from this study were 652 parents (average age range = 35–44; 65.0% female). To be eligible, parents had to have children who were between the ages of 3–6 years who were in preschool and kindergarten. They also had to pass all twelve attention checks during the report. Parents’ highest level of education ranged from some high school (1.5%) to a doctoral or advanced degree (7%), with the average response being a four-year degree (33%). Parents’ ethnicity is as follows: 87.4% were White, 6.0% were Black or African American, 3.1% were Bi or Multi-Racial, and 2.0% were Asian. Less than 1 % of the sample identified as American Indian or Alaska Native, Native Hawaiian or Pacific Islander, or other race or ethnicity. Additionally, 8.6% of the participants identified as Hispanic. The average annual household income was $70,000–$79,999, with 2.5% reporting less than $10,000 and 12% reporting more than $150,000.

#### Measures

##### Parent communalism

The Parent Communalism measure (Boykin et al., 1997; Grayman-Simpson and Mattis, 2017) was a seven-item self-report questionnaire aimed to assess the extent that parental participants believed in social relations, concern for others, and group effort. For example, the questionnaire assessed the extent that parents believed the following statement is true: “I believe that when people are close to one another (like family and friends) they should be accountable for each other’s welfare.” on a scale from 1 = Completely false to 6 = Completely true (alpha = 0.79).

##### Home math environment

The Home Math Environment measure (Hart et al., 2016; Zippert et al., 2020) is a 124-item survey designed with two subscales to be completed by a parent or guardian. The first subscale assessed how often parents or guardians participated in certain mathematical activities with their child. For example, “In the PAST MONTH, how often did you and your child engage in the following? - Count objects” on a scale from 1 = Never to 6 = Multiple times a day (*α* = 0.95). The second subscale assessed the child’s level of difficulty when engaging in the activity. For example, “This activity is ___ for my child,” in regards to Counting Objects on a rating scale from 1 = Too easy to 3 = Too hard (*α* = 0.95). Due to the length of the survey, there were four attention checks integrated within this measure. Only parents or guardians who passed all four attention checks were included in this study.

##### Math skills development

To assess children’s math skills, we utilized parental reports using two measures: (1) how high children can consistently and accurately count up to 100 (number count; *M* = 44) and (2) the count of numbers children can identify from 0 to 15 (number identification; *M* = 12). Means on the number count measure were 19.26 (*SD* = 19), 34.71 (*SD* = 32.51), 64.19 (*SD* = 38.26), and 75.29 (*SD* = 35.92) for three- through six-year-olds, respectively. Means on the number identification measure were 9.85 (*SD* = 5.27), 11.28 (*SD* = 5.01), 13.92 (*SD* = 4.13), and 13.44 (*SD* = 5.11) for three- through six-year-olds, respectively.

##### Covariates

Parents reported children’s gender (49% male) and age (*M* = 4.25) as individual-level covariates. They also reported their own race, ethnicity, highest level of education, and median annual household income. Gender is coded as 1 = female and 0 = male for children and parent or guardians. Parents or guardians reported their children’s age in years at the time of data collection (i.e., between December 2021 and January 2022).

### Data analytic strategy

#### Factor analysis

To determine the best fitting model of the HME factor structure, a series of six confirmatory factor models were conducted in Mplus (Muthén and Muthén, 2017), similar to prior investigations of the HME factor structure (Hart et al., 2016; Purpura et al., 2020). Prior to the analyses, items for which more than one-third of participants responded “never” were dropped. As a result, 31 items were excluded from all analyses (Table 1). The remaining 29 items were included in the next step.

**Table 1:** Home math environment items, response rates, and descriptive statistics.

Item #	Item description	% of “Never” responses	M	SD
1	Count objects	2	4.87	1.21
2	Count down	10	3.87	1.55
3	Identify names of written numbers	8	4.17	1.50
4	Print numbers	15	3.44	1.54
5	Use calendars and dates	29	2.85	1.61
6	Connect-the-dot activities	27	2.59	1.40
7	Use number activity books	16	3.10	1.40
8	Read number storybooks	17	3.17	1.48
9	Play board games	25	2.61	1.30
10	Learn simple sums	28	3.02	1.65
11	Sort things by size, color, or shape	5	4.16	1.46
12	Make collections	22	3.07	1.61
13	Recite numbers in order	3	4.80	1.26
14	Guess the number of things	21	3.12	1.59
15	Note numbers on signs	14	3.62	1.55
16	Interact with clocks	27	3.03	1.67
17	Use numbers in reference to temperature, time, dates	30	3.10	1.77
18	Compare sizes of numbers	22	3.27	1.64
19	Play with puzzles	6	3.49	1.31
20	Play with LEGO	9	3.77	1.47
21	Play with blocks	6	4.12	1.43
22	Make patterns with objects or sounds	19	3.26	1.61
23	Figure out what comes next in a pattern	19	3.16	1.54
24	Watch TV shows or videos what show and talk about patterns	18	3.12	1.49
25	Read books that show or talk about patterns	17	3.07	1.45
26	Play computer games, apps, or visit interactive websites that include pattern games	30	2.81	1.55
27	Play hand or movement games that involve patterns	32	2.57	1.47
28	Compare groups of objects to identify more/less or same/equal	24	2.80	1.43
29	Compare one object by directly comparing them to another	10	3.74	1.51
Items not included in the factor analysis				
30	Use number or arithmetic flashcards	40	2.41	1.48
31	Measure ingredients when cooking	33	2.43	1.35
32	Being timed	35	2.82	1.68
33	Play with calculators	60	1.78	1.21
34	Play card games	36	2.31	1.32
35	Sing math songs	48	2.31	1.59
36	Keep track of money	38	2.31	1.38
37	Play games in the car that involve math	47	2.10	1.33
38	Use computer/video games to do math	36	2.65	1.56
39	Do word problems	62	1.85	1.29
40	Help with math homework	60	2.10	1.53
41	Measure lengths/widths	54	1.85	1.16
42	Use computer to draw or play with shapes	36	2.63	1.60
43	Use computer for spatial games	60	1.93	1.39
44	Draw maps	72	1.46	0.89
45	Draw plans for buildings	83	1.31	0.81
46	Use kits to build models	51	1.78	1.02
47	Fold or cut paper to make 3D objects	58	1.71	1.03
48	Wear a watch	70	1.64	1.20
49	Play with an abacus	81	1.35	0.89
50	Play with dominoes	67	1.52	0.91
51	Use scales	70	1.50	0.91
52	Play with math mat	81	1.34	0.81
53	Talk about math in reference to sports	75	1.46	0.93
54	Do math in your head	50	2.34	1.63
55	Play with number fridge magnets	41	2.60	1.70
56	Count out money	37	2.25	1.29
57	Describe patterns in words	36	2.47	1.45
58	Copy a pattern with different materials	41	2.30	1.42
59	Discuss patterns in days of the week, months of the year, or seasons	33	2.49	1.43
60	Use a ruler or other objects to measure and discuss length	54	1.83	1.12

In the first model, one factor included the remaining 29 items to represent the diversity of possible home math activities. Second, two unspecified factors were fitted, followed by the fit of two specific factors (direct and indirect). In the fourth model, three factors were specified: direct, indirect, and patterns. The last two models fit a bifactor model that included a general factor of all items, as well as specific factors of direct, indirect (model 5), and patterns (model 6). Model fit was compared using fit statistics indices including the Chi-square (𝜒^2^), Chi-square/Degrees of Freedom Ratio (𝜒^2^/df), Akaike Information Criterion (AIC), sample-size adjusted Bayesian Information Criterion (SABIC), Root Mean Square Error of Approximation (RMSEA), Standardized Root-Mean-Square residual (SRMR), Comparative Fit Index (CFI), and Tucker-Lewis Index (TLI). The 𝜒^2^ greater than 0.05 indicated there was no significant difference between models. The 𝜒^2^/df statistic, an additional statistic which accounts for the complexity of the model with less sensitivity to sample size, indicated reasonable fit within the range of 2–5 (Sathyanarayana and Mohanasundaram, 2024). Lower AIC and SABIC scores were indicators of a better model fit (Burns et al., 2022; Ferguson et al., 2020). The RMSEA is recommended to fall between 0.05 and 0.08 (Browne and Cudeck, 1992), while the SRMR equal to or below 0.08 was indicative of a better fit. Both the CFT & TLI are recommended to be above 0.95 (Hu and Bentler, 1999).

As shown in Table 2, none of the confirmatory factor analysis models indicated a best-fit for the data. In the third step, an exploratory factor analysis was conducted on the 29 items to establish the best model fit (Table 3). A Varimax Orthogonal rotational method was used to analyze the data such that the specific factors were uncorrelated with the general factor and also uncorrelated with each other. Factors with eigenvalues greater than 1 were extracted and the model fit was evaluated using the above criteria. Once the number of factors was determined, items were removed from further analysis if they failed to load onto a factor less than 0.40 or cross-loaded on two or more factors more than 0.40.

**Table 2:** Model fit indices for the home math environment — confirmatory factor analyses.

Model	Description	𝜒^2^, (df)	AIC	SABIC	RMSEA 90% CI	CFI	TLI	SRMR
1	1 factor	2996.15 (377)	49133.1	49246.64	0.10 (0.100, 0.107)	0.64	0.61	0.09
2	2 factors (unspecified)	1895.99 (349)	48088.94	48239.02	0.08 (0.079, 0.086)	0.79	0.75	0.06
3	2 factors (D, I)	288.81 (376)	48967.76	49082.6	0.10 (0.097, 0.103)	0.66	0.64	0.09
4	3 factors (D, I, P)	2690.404 (374)	48833.36	48950.81	0.10 (0.094, 0.101)	0.68	0.66	0.08
5	Bifactor (General; D, I)	1876.314 (348)	48071.27	48222.65	0.08 (0.078, 0.086)	0.79	0.76	0.08
6	Bifactor (General; D, I, P)	1837.85 (348)	48032.8	48184.19	0.08 (0.077, 0.085)	0.80	0.76	0.07

Factor Descriptions: D = Direct skills; I = Indirect skills; P = Pattern skills. All Chi-square (𝜒^2^) and Root Mean Square Error of Approximation (RMSEA) results were significant at *p* = < 0.001. AIC = Akaike Information Criterion; SABIC = sample-size adjusted Bayesian Information Criterion; SRMR = Standardized Root Mean Square residual; CFI = Comparative Fit Index; TLI = Tucker-Lewis Index.

**Table 3:** Model fit indices for the home math environment — exploratory factor analyses.

Model	% total variance	𝜒^2^, df	AIC	SABIC	RMSEA 90% CI	CFI	TLI	SRMR
1	31%	2996.15 (377)	49133.10	49246.64	0.10 (0.100, 0.107)	0.64	0.61	0.09
2	40%	1895.99 (349)	48088.94	48239.02	0.08 (0.079, 0.086)	0.79	0.75	0.06
3	46%	1429.34 (322)	47676.29	47861.61	0.07 (0.069, 0.076)	0.85	0.81	0.05
4	50%	1121.35 (296)	47420.30	47639.55	0.07 (0.061, 0.069)	0.89	0.85	0.04
**5**	**54%**	**863.25** **(271)**	**47212.2**	**47464.08**	**0.06** **(0.054, 0.062)**	**0.92**	**0.87**	**0.03**
6	58%	674.62 (247)	47071.57	47354.76	0.05 (0.047, 0.056)	0.94	0.90	0.03
7	61%	524.24 (224)	46967.19	47280.40	0.05 (0.040, 0.050)	0.96	0.93	0.03
8	64%	426.14 (202)	46913.09	47255.02	0.04 (0.036, 0.047)	0.97	0.94	0.02
9	67%		46901.78	47271.11	0.05	0.96	0.92	0.02

AIC = Akaike Information Criterion; SABIC = sample-size adjusted Bayesian Information Criterion; SRMR = Standardized Root Mean Square residual; CFI = Comparative Fit Index; TLI = Tucker-Lewis Index. Bold values indicate best model fit.

#### Mediation analysis

The regression and mediation analyses were conducted using IBM SPSS Statistics (Version 28; IBM Corp, 2021). Overall HME and the five HME factors, both frequency and difficulty, were used as the independent variable. Children’s math performance was the dependent variable, which included how high a child could count and the sum of all numbers a child could identify. Parents’ communalism was the mediating variable.

First, simple regressions of HME on communalism and both measures of math performance were analyzed, as well as the regression of communalism on both measures of math performance. Then, the PROCESS macro in SPSS created by Hayes (2022) was used to determine the indirect effect of HME on children’s math performance through parental communalism beliefs. PROCESS gives a summary of the model, including the regressions between variables, as well as the direct and indirect effects of the independent variable on the dependent variable. This mediation analysis used the same independent and dependent variables as the regression analysis, but communalism was listed as the mediating variable.

After the initial analysis was conducted, a second mediation analysis was done using two covariates: whether the parent was Hispanic and the highest household education level. The two covariates were chosen due to preliminary analyses showing significant differences between levels in terms of HME or communalism scores (see Results).

## Results

### Factor analysis

The first aim of this study was to examine the factor structure of the home math environment. The six confirmatory factor analyses (1-factor, two 2-factor, 3-factor, two bifactor models) resulted in poor model fit according to the fit indices (Table 2). Of the six exploratory factor analyses, models 2, 5, and 6 resulted in the strongest fit indices. Although the eigenvalue and evaluation of the fit indices revealed a six-factor solution, examination of the factor structure revealed there were no HME items on the sixth factor with loadings greater than 0.40. The five-factor solution was selected as the preferred model. The Chi-square statistic indicated a significant difference between models (𝜒^2^ (271) = 863.25, *p* < 0.001) and the Chi-square/Degrees of Freedom Ratio indicated reasonable fit (𝜒^2^/df = 3.19). Akaike Information Criterion (AIC) and sample-size adjusted Bayesian Information Criterion (SABIC) values were lower when compared to earlier models (AIC = 47212.20 and SABIC = 47464.08). The Root Mean Square Error of Approximation (RMSEA) value (0.06) was at the cutoff for reasonable fit. The Standardized Root-Mean-Square residual value (0.03) was well below the cutoff which indicated good fit. While the Tucker-Lewis Index (TLI) value (0.88) was below the cut-off for good fit, the Comparative Fit Index (CFI) value (0.92) was within the range for acceptable fit.

As found in Table 4, the final model included 21 items representing the five factors. Factor 1 was comprised of four Numeracy and Counting items: counting objects, counting down, identifying names of written numbers, and reciting numbers in order (*α* = 0.77). Effect sizes in Factor 1 ranged from 0.51–0.78 indicated moderate to strong effects. Factor 2 indicated Numeracy Activities with three items: connect the dot activities, number activity books, and number storybooks (*α* = 0.70). Effect sizes for Factor 2 ranged from minimal to moderate (0.45–0.67). Factor 3 was comprised on seven Numeracy Application activities: print numbers, use of calendars and dates, playing board games, learning simple sums, interacting with clocks, using numbers to read temperature, time, or dates, comparing sizes of numbers (*α* = 0.84). Effect sizes for Factor 3 ranged from minimal to strong (0.48–0.73). Factor 4 indicated Pattern activities: making patterns with objects and sounds, figuring out what comes next in a pattern, comparing groups of objects to identify more/less or same/equal, and comparing one object directly to another (*α* = 0.81). Effect sizes for Factor 4 ranged from 0.53–0.67 which indicated moderate effects. Factor 5 was comprised of three items indicating Patterns in Media: watching tv or video focused on patterns, reading books that show or talk about patterns, and playing computer games/apps/websites that include pattern games (α = 0.72). Effect sizes for Factor 5 ranged from minimal to strong (0.42–0.90).

**Table 4:** Eigenvalues and standardized factor loadings for the 5-factor model.

Eigenvalue 1	8.85
Eigenvalue 2	2.71
Eigenvalue 3	1.67
Eigenvalue 4	1.33
Eigenvalue 5	1.19
Eigenvalue 6	1.04

Factor loadings					
	1	2	3	4	5
Factor 1: Numeracy/Counting M = 4.43, SD = 1.07, Alpha = 0.77					
1	0.757*	0.095*	0.092*	0.194*	0.117*
2	0.510*	0.213*	0.264*	0.054	0.221*
3	0.543*	0.211*	0.274*	0.121*	0.076*
13	0.651*	0.088*	0.124*	0.256*	0.106*
Factor 2: Numeracy Activities M = 2.95, SD = 1.13, Alpha = 0.70					
6	0.124*	0.541*	0.319*	0.180*	0.143*
7	0.224*	0.673*	0.209*	0.149*	0.080*
8	0.261*	0.449*	0.079*	0.190*	0.182*
Factor 3: Numeracy Application M = 3.05, SD = 1.14, Alpha = 0.84					
4	0.298*	0.394*	0.488*	0.043	−0.061
5	0.090*	0.197*	0.650*	0.048	0.112*
9	0.013	0.305*	0.478*	0.095*	0.091*
10	0.100*	0.254*	0.664*	0.032	0.012
16	0.089*	0.122*	0.633*	0.145*	0.016
17	0.053	−0.036	0.734*	0.111*	−0.010
18	0.215*	0.037	0.599*	0.373*	0.078*
Factor 4: Patterns M = 3.23, SD = 1.22, Alpha = 0.81					
22	0.304*	0.158*	0.076*	0.609*	0.254*
23	0.177*	0.201*	0.323*	0.528*	0.236*
28	0.152*	0.098*	0.291*	0.582*	0.125*
29	0.234*	0.051	0.158*	0.667*	0.204*
Factor 5: Patterns in Media M = 3.00, SD = 1.20, Alpha = 0.72					
24	0.104*	0.032	−0.005	0.118*	0.902*
25	0.170*	0.113*	0.077*	0.279*	0.616*
26	−0.011	0.194*	0.238*	0.297*	0.420*

* indicates *p* < 0.05. Bold values indicate the factor loading.

The factors have low to moderate correlations ranging from 0.25–0.51 (Table 5). The weakest correlation was found between Numeracy Applications and Patterns in Media. The strongest correlations were between (1) Numeracy/Counting and Patterns and (2) Patterns and Patterns in the Media. All correlations were statistically significant (*p* < 0.001).

**Table 5:** Home math environment correlations.

Factor	1	2	3	4	5
1. Numeracy/Counting	—				
2. Numeracy Activities	0.47	—			
3. Numeracy Applications	0.44	0.49	—		
4. Patterns	0.51	0.44	0.45	—	
5. Patterns in Media	0.34	0.36	0.25	0.51	—

All correlations are significant, *p* < 0.001.

### Preliminary analyses

Examined the general performance of children and their families considering their home math environment, communal beliefs espoused in the home, math skill development, and demographic characteristics such as age, gender, race, ethnicity, parental education, and household income.

#### Home math environment (frequency)

The frequency of home math engagement (*M* = 3.33, *SE* = 0.03) was significantly different for children based on their age, *F* (3, 648) = 5.98, *p* <. 001, *η*^2^ = 0.027. For the general home math environment, four- through six-year-olds were engaged in math activities above average (*M* = 3.38, 3.45, and 3.38, respectively). Bonferroni-adjusted pairwise comparisons indicated four-year-olds experienced significantly higher frequency in home math when compared to three-year-olds, as the interval did not include zero, 95% *CI* [0.03, 0.50]. Similarly, five-year-olds experienced higher frequency in the home math environment when compared to three-year-olds, as the interval did not include zero, 95% *CI* [0.12, 0.56]. No other age differences were statistically significant. The frequency of home math engagement was not significantly different for children based on their gender or their parent or guardian’s race, ethnicity, education, or household income.

We examined differences in the frequency of certain types of home math engagement. Findings indicated significant age differences in Numeracy/Counting, *F* (3, 648) = 4.40, *p* = 0.005, *η*^2^ = 0.02. Using four-year-olds as the reference group with the highest numeracy and counting frequency (*M* = 4.60, *SE* = 0.07), they had significantly higher engagement when compared to six-year-olds (*M* = 4.06, *SE* = 0.15), 95% CI [−0.93, −0.14]. There were significant age differences in Numeracy Activities, *F* (3, 648) = 3.30, *p* = 0.020, *η*^2^ = 0.015. Four-year-olds also had higher frequency of numeracy activities in the home environment, (*M* = 3.15, *SE* = 0.08) when compared to six-year-olds (*M* = 2.73, *SE* = 0.13), 95% CI [−0.84, −0.01].

We also found significant age differences in Numeracy Applications, *F* (3, 648) = 51.17, *p* < 0.001, *η*^2^ = 0.19. Using six-year-olds as the reference group with the highest frequency in numeracy applications (*M* = 3.66, *SE* = 0.11), they had significantly higher engagement in the home environment when compared to three-year-olds (*M* = 2.35, *SE* = 0.07), 95% CI [0.93, 1.69]; and four-year-olds (*M* = 3.01, *SE* = 0.08), 95% CI [0.27, 1.03]. Lastly, there were significant age differences in Patterns in Media, *F* (3, 648) = 3.16, *p* = 024, *η*^2^ = 0.01. Using three-year-olds as the reference group with the highest frequency in Patterns in Media (*M* = 3.21, *SE* = 0.09), they had significantly higher frequency when compared to five-year-olds (*M* = 2.90, *SE* = 0.08), 95% CI [−0.63, −0.01].

#### Home math environment (difficulty)

Parents’ perceptions of children’s difficulty when engaging in home math (*M* = 2.08, *SE* = 0.01) was significantly different based on child age, *F* (3, 648) = 88.27, *p* <. 001, *η*^2^ = 0.029. Three-year-olds served as the reference group with the highest general home math environment scores, (*M* = 2.31, SE = 0.02). We found they significantly experienced difficulty more than four-year-olds (*M* = 2.13, *SE* = 0.02), 95% CI [−0.26, −0.11]; five-year-olds (*M* = 1.92, *SE* = 0.02), 95% CI [−0.47, −0.32]; and six-year-olds (*M* = 1.86, *SE* = 0.03), 95% CI [−0.55, −0.35].

Considering the various types of home math engagement, the findings were consistent. Three-year-olds were perceived as experiencing more difficulty while engaging in home math (i.e., counting, numeracy activities, numeracy applications, patterning, and patterning within media). It was also consistently found that this was a significant difference when comparing three-year-olds with all other age groups, with one exception. For Patterning in the Media (*M* = 2.06, *SD* = 0.41), three-year-olds (*M* = 2.16, *SE* = 0.03) experienced significantly more difficulty when compared to five-year-olds (*M* = 1.97, *SE* = 0.03), 95% CI [−0.30, −0.09]; and six-year-olds (*M* = 1.91, *SE* = 0.05), 95% CI [−0.40, −0.11].

#### Parental communal beliefs

Parent or guardian communal beliefs (*M* = 3.77, *SD* = 0.84) were significantly different based on ethnicity, *F* (1, 650) = 4.95, *p* = 0.026, *η*2 = 0.01. Parents who identified as Hispanic or Latine were more likely to hold communal beliefs (*M* = 4.01, *SE* = 0.10). Child age, child gender, parent/guardian race, parent/guardian highest level of education, and household income were not significantly associated with parental communal beliefs.

#### Children’s math skill development

Not surprisingly, age was a significant factor in children’s counting (*M* = 44.34, *SD* = 28.12, *F* (3, 648) = 95.98, *p* < 0.001, *η*2 = 0.31) and number identification (*M* = 11.96, *SD* = 5.12, *F* (3, 648) = 27.53, *η*2 = 0.11). Six-year-olds, the reference group with the highest counting skills (*M* = 75.29, *SE* = 4.23), performed significantly higher when compared to three-year-olds (*M* = 19.26, *SE* = 1.37), 95% CI [44.41, 67.65]; and four-year-olds (*M* = 34.71, *SE* = 2.48), 95% CI [28.77, 52.39]. No other demographic characteristics were associated with children’s ability to count consistently and accurately from 0 to 100.

There were significant age differences in children’s number identification such that five-year-olds (reference group with the highest score, *M* = 13.92, *SE* = 0.28) outperformed three-year-olds (*M* = 9.85, *SE* = 0.38), 95% CI [2.81, 5.34]; and four-year-olds (*M* = 11.28, *SE* = 0.38), 95% CI [1.33, 3.95]. No other demographic characteristics were significantly associated with children’s number identification.

### Regression analysis

Simple regression analyses were used to determine the relation between home math environment, communalism, and math performance. Home math environment scores (frequency) did not significantly predict communalism scores, *β* = 0.041, *t* (650) = 1.058, *p* = 0.291.

Home math environment scores (difficulty) also did not significantly predict communalism scores, *β* = 0.033, *t* (650) = 0.851, *p* = 0.395. Communalism scores did not significantly predict how high a child could count, *β* = −0.067, *t* (650) = −1.700, *p* = 0.090, or how many numerals a child could identify, *β* = −0.032, *t* (650) = −0.816, *p* = 0.415. However, home math environment scores (frequency) predicted how high a child could count, *β* = 0.227, *t* (650) = 5.944, *p* < 0.001, and how many numerals a child could identify, *β* = 0.250, *t* (650) = 6.579, *p* < 0.001. Home math environment scores (difficulty) also predicted how high a child could count, *β* = −0.598, *t* (650) = −19.046, *p* < 0.001, and how many numerals a child could identify, *β* = −0.435, *t* (650) = −12.312, *p* < 0.001 (Table 6).

**Table 6:** Regression analysis of home math environment on children’s math abilities.

Home math environment		How high a child can count	Sum of numbers a child can identify
Numeracy/Counting	Frequency	0.07	0.17*
	Difficulty	−0.55*	−0.48*
Numeracy Activities	Frequency	0.05	0.18*
	Difficulty	−0.40*	−0.35*
Numeracy Applications	Frequency	0.43*	0.35*
	Difficulty	−0.60*	−0.39*
Patterns	Frequency	0.04	0.08
	Difficulty	−0.35*	−0.22*
Patterns in Media	Frequency	−0.01	0.003
	Difficulty	−0.25*	−0.14*
Overall	Frequency	0.23*	0.25*
	Difficulty	−0.60*	−0.44*

*β* coefficients, * indicates *p* < 0.001.

Another regression analysis was used to determine whether the HME factors chosen for the confirmatory factor analysis predicted communalism and math performance. The only factor that predicted communalism scores was Numeracy Activities (frequency), *β* = 0.095, *t* (650) = 2.42, *p* = 0.016. The factors that predicted how high a child could count were: Counting and Numeracy (difficulty), *β* = −0.548, *t* (650) = −16.722, *p* < 0.001; Numeracy Activities (difficulty), *β* = 0.095, *t* (650) = −11.203, *p* < 0.001; Numeracy Application (frequency), *β* = 0.430, *t* (650) = 12.150, *p* < 0.001; Numeracy Application (difficulty), *β* = −0.599, *t* (650) = −19.093, *p* < 0.001; Patterns (difficulty), *β* = −0.354, *t* (650) = −9.635, *p* < 0.001; and Patterns in Media (difficulty), *β* = −0.247, *t* (650) = −6.492, *p* < 0.001. The factors that predicted how many numerals a child could identify were: Counting and Numeracy (frequency), *β* = 0.167, *t* (650) = 4.320, *p* < 0.001; Counting and Numeracy (difficulty), *β* = −0.475, *t* (650) = −13.750, *p* < 0.001; Numeracy Activities (frequency), *β* = 0.179, *t* (650) = 4.627, *p* < 0.001; Numeracy Activities (difficulty), *β* = −0.354, *t* (650) = −9.648, *p* < 0.001; Numeracy Application (frequency), *β* = 0.349, *t* (650) = 9.499, *p* < 0.001; Numeracy Application (difficulty), *β* = −0.394, *t* (650) = −10.916, *p* < 0.001; Patterns (difficulty), *β* = −0.222, *t* (650) = −5.813, *p* < 0.001; and Patterns in Media (difficulty), *β* = −0.140, *t* (650) = −3.612, *p* < 0.001.

### Mediation analysis

A mediation analysis was conducted with the PROCESS macro in SPSS (Hayes, 2022) to determine whether communalism mediates the relationship between home math environment and math performance. As the results of the regression analyses indicated, there was no significant indirect effect of home math environment (frequency) on how high a child could count through communalism scores (effect = −0.0067, *SE* = 0.0082, 95% CI [−0.0266, 0.0069]), and no significant indirect effect of home math environment (difficulty) on how high a child could count through communalism scores (effect = −0.0087, *SE* = 0.014, 95% CI [−0.042, 0.0155]). Similarly, there was no significant indirect effect of home math environment (frequency) scores on how many numerals a child could identify through communalism scores (effect = −0.0005, *SE* = 0.0008, 95% CI [−0.0024, 0.0007]), and no significant indirect effect of home math environment (difficulty) scores on how many numerals a child could identify through communalism scores (effect = −0.0004, *SE* = 0.0014, 95% CI [−0.0038, 0.0023]). No significant indirect effect was found for any of the individual HME factors.

**Figure 1:**
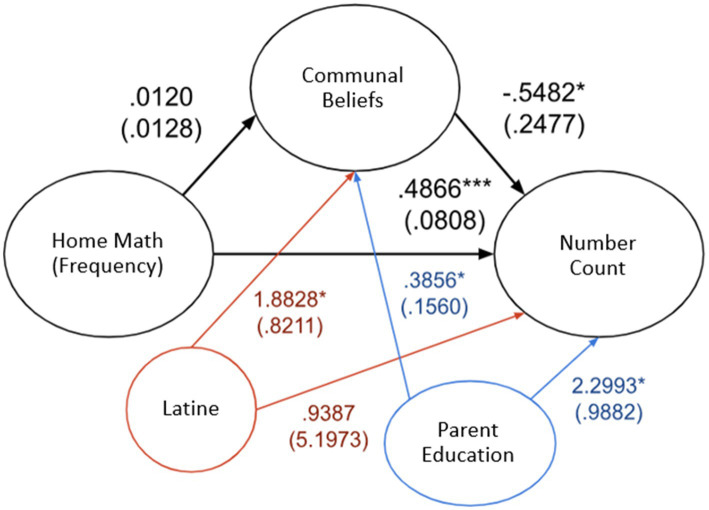
Direct effect between home math environment (frequency) and child’s numeracy/counting. Covariates include Latine = Parent identified as Latine and Parent Education = highest parental education. Effect (SE), *p < 0.05, **p < 0.01, ***p < 0.001.

**Figure 2:**
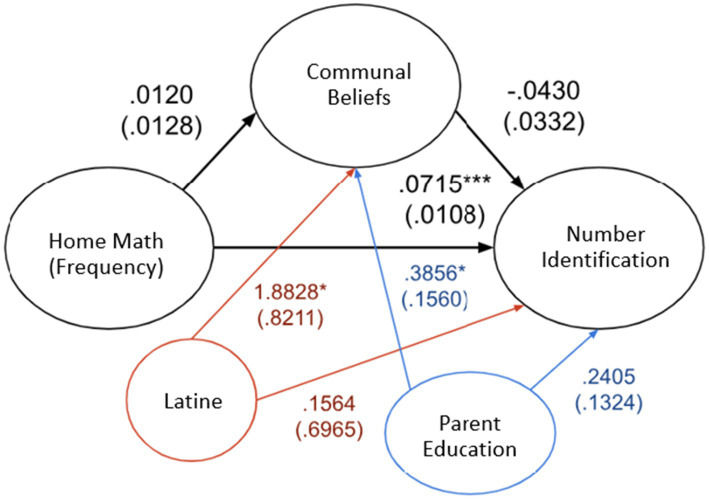
Direct effect between home math environment (frequency) and child’s number identification. Covariates include Latine = Parent identified as Latine and Parent Education = highest parental education. Effect (SE), *p < 0.05, **p < 0.01, ***p < 0.001.

A second mediation analysis was conducted by adding two variables as covariates: the highest education level in the household and whether the parent identified as Hispanic. As illustrated by Figure 1, the new analysis revealed similar results; there was no significant indirect effect of home math environment (frequency) on how high a child could count through communalism scores (effect = −0.0066, *SE* = 0.0041, 95% CI [−0.0126, 0.0036]), and no significant indirect effect of home math environment (difficulty) on how high a child could count through communalism scores (effect = −0.0020, SE = 0.0028, 95% CI [−0.0084; 0.0029]). Similarly, there was no significant indirect effect of home math environment (frequency) scores on how many numerals a child could identify through communalism scores (effect = −0.0005, SE = 0.0008, 95% CI [−0.0024, 0.0008]; Figure 2), and no significant indirect effect of home math environment (difficulty) scores on how many numerals a child could identify through communalism scores (effect = −0.0007, SE = 0.0016, 95% CI [−0.0044; 0.0022]). No significant indirect effect was found for any of the individual HME factors.

## Discussion

This study examined the relation between parental communal beliefs, home math engagement, children’s math skill development, and whether communal beliefs mediated these relations. The exploration of home communal orientation expands upon the prior work within school contexts that found a positive association between children’s home communal orientation and their mathematics performance. While our findings did not indicate communalism as a mediating factor, it could be useful to consider it as a factor in the home mathematics environment, particularly for children with certain demographic characteristics. The results also suggest that the home mathematics environment can be conceptualized differently than prior research suggests, with an inclusion of non-numeracy domains that are often misinterpreted or missing from examination (Hornburg et al., 2021), and with more specific focus on the types of math activities. Due to the predictive nature of these home mathematics activities on children’s numeral identification and counting, this study provides a step forward for considering how the frequency and/or difficulty of these activities influence mathematics skill development.

### Factor structure of the home math environment

Beyond the conceptualization of the home math environment, including formal, informal, and spatial activities (Dearing et al., 2012; Hart et al., 2016; LeFevre et al., 2009), our findings suggested five distinct factors that comprise home math engagement. These included numeracy and counting items (e.g., counting down), numeracy activities (e.g., connect the dot activities), numeracy application (e.g., compare size of numbers, *α* = 0.84), patterning (e.g., next in the pattern), and patterns involving media (e.g., computer patterns). We also found evidence that parent or guardian’s use of these activities varied based on the child’s age, such that numeracy and counting, numeracy activities, patterning, and patterning using media were more frequent in households with younger preschool-aged children. Numeracy application was more frequent in households with older preschool-aged children. This updated conceptualization of the home math environment can help researchers better examine the relation between children’s developmental trajectory (Ehrman et al., 2023) and the home math environment generally, as well as the appropriateness of specific activities based on age.

### Home math environment predicting math skill development

Our findings support a positive and significant relation between the home math environment and children’s math skill development. Regarding the development of children’s numeracy skills, the application of numeracy skills (i.e., playing board games, interacting with clocks, and comparing the sizes of numbers) was associated with higher skill development. The frequency of home math engagement matters such that reports of higher frequency were consistently associated with higher math skill development. For most of the math activities, parents’ reports of lower levels of difficulty was associated with higher numeracy skills. The exception was the report of numeracy activities for which there was a positive correlation. Activities such as connect the dot activities, number activity books, and number storybooks, though challenging for children to complete, were associated with positive skill development.

In the examination of children’s number identification skills, we found that lower levels of difficulty in all of the home math activity domains were associated with higher skill development. It is likely that parent’s perceptions of task/activity difficulty varied based upon the specific activity in question. It would be useful to consider the constructs of difficulty and age-appropriateness to determine whether these are similar or distinct within the home math environment, and how they can inform the measures and practices of researchers and practitioners.

Higher frequency of counting exercises, numeracy activities, and number application were associated with higher skill development as well. These findings were supported by literature in the area of family mathematics activities in which 87% of studies found a relation between the home math environment and children’s outcomes (Eason et al., 2022). The conceptualization of home math domains will allow future research to focus on specific mathematics activities beyond the broad constructions of direct or formal, indirect or informal, and spatial activities.

### The role of communalism

Communal beliefs, the value of social connectedness and social interdependence that leads to aims such as helping people and working collaboratively with others (Dasgupta et al., 2022; McElveen, 2024), have historical and cultural roots in minority communities (i.e., African American, Asian, Latine; Rucker et al., 2018); thus it was not surprising that Hispanic or Latine parents or guardians reported higher communalism. We found that among all parents or guardians, college graduates and postgraduates reported higher communal beliefs than those without a four-year degree. This finding is inconsistent with previous research that suggested that individuals with higher socioeconomic status (i.e., status based on education or wealth) tend to exhibit more agency and individualism. This inconsistency may be explained by the situational nature of the questions and their concern for family and social groups. This could also be interpreted as individuals with lower education levels reporting greater agency or individualism as a mechanism for focusing on themselves and their own advancement (Rucker et al., 2018). Another explanation of this inconsistency could be derived from the differences in measurement of socioeconomic status across studies. Prior research used similar measures of socioeconomic status (i.e., education and income separately) while other have varied in the use of categorical vs. continuous variables (Daucourt et al., 2021) and composites created to represent social position (Levine et al., 2010). There is more to be explored in this area of research and understanding the association between communal beliefs and socioeconomic status through the utilization of quantitative and qualitative methodology could be useful knowledge for researchers and educators alike.

When examining the relation between communal beliefs and the home math environment, we found parents or guardians with higher communal beliefs engaged more frequently in numeracy activities. Making connections between the data, these are the same activities parents found more difficult for their children. This could indicate an instance of scaffolded learning and/or demonstrates a potential benefit of communal learning, however, more research is necessary to substantiate this link. Prior work on the role of communalism in the development of minority children’s math performance (Coleman et al., 2017) would point to its mediating role for younger children. However, with such a low sample of minoritized children in this sample, additional examinations with more diverse samples of families would be warranted.

### Limitations and future directions

There are a few limitations to note that lead to important future directions for better understanding the home math environment, its relation to children’s math skill development, and cultural beliefs as a contributing factor. First, as indicated, our sample primarily consisted of White and higher-income families. While it is important to understand these constructs among all families regardless of race and socioeconomic status, we also acknowledge the importance of specifically understanding race and socioeconomic status for the purpose of improving equitable outcomes for minoritized preschoolers. Additional research with more diverse sample populations (i.e., Latine, Black, and Asian families) is key to future research.

We acknowledge the limitation of the communal orientation measure. While the measure allowed for the understanding of families’ social orientation generally, it was not targeted toward the home math environment. Future studies should include additional measures to better understand the direct influence of social orientation on parent–child math-focused interactions, possibly through the triangulation of parent report and observational data. While this is an emerging area of study, there are communal measures (Coleman et al., 2023; Matthews et al., 2021; McElveen, 2024) from which items could be adapted. It would also be important to gather information about the communal aspects of the home math environment beyond parent–child interaction, such as sibling interactions.

Another limitation of this study was the measure of children’s early mathematics skills. The use of parent-reported measures did not provide an opportunity for researchers to independently assess children’s mathematics skills. In addition, parents reported on a limited range of mathematics skills instead of the broad range of skills assessed in the home numeracy measure. Future research should triangulate parent reports with robust measures of children’s mathematics skills to strengthen the research design and its findings.

This study provided important insights into the structure of the home mathematics environment and comprehensively assessed the frequency and difficulty of home math activities to understand the role of both constructs in the development of children’s early math skills. This study also explored the sociocultural construct of communalism which has been more widely examined in the formal (i.e., school) learning environment to understand its role in the informal learning environment for preschool children. Based on our findings, the contributions of the home math environment vary based on the specific type of activity, its frequency, and the difficulty of math activities that children are exposed to. These findings provide support for future research to further assess these relations across a diverse sample of children and their families using robust measures of mathematical skills and communal interactions inclusive of the family structure. The findings of this and future research provide an opportunity to consider social orientation and social interactions in the research and practice of math learning in the home environment to foster children’s strong performance in future math.

## Data Availability

Publicly available datasets were analyzed in this study. This data can be found at: https://doi.org/10.33009/ldbase.1647978201.2f65.
